# Genomic Patterns of Homozygosity in Chinese Local Cattle

**DOI:** 10.1038/s41598-019-53274-3

**Published:** 2019-11-18

**Authors:** Lingyang Xu, Guoyao Zhao, Liu Yang, Bo Zhu, Yan Chen, Lupei Zhang, Xue Gao, Huijiang Gao, George E. Liu, Junya Li

**Affiliations:** 1 Innovation Team of Cattle Genetic Breeding, Institute of Animal Sciences, Chinese Academy of Agricultural Sciences, Beijing, 100193 China; 2 Animal Genomics and Improvement Laboratory, Department of Agriculture-Agricultural Research Services, Beltsville, Maryland 20705 United States of America

**Keywords:** Genomics, Animal breeding

## Abstract

Genome-wide single nucleotide polymorphism (SNP) arrays can be used to explore homozygosity segments, where two haplotypes inherited from the parents are identical. In this study, we identified a total of 27,358 runs of homozygosity (ROH) with an average of 153 ROH events per animal in Chinese local cattle. The sizes of ROH events varied considerably ranging from 0.5 to 66 Mb, with an average length of 1.22 Mb. The highest average proportion of the genome covered by ROH (~11.54% of the cattle genome) was found in Nanda cattle (NDC) from South China, whereas the lowest average proportion (~3.1%) was observed in Yanhuang cattle (YHC). The average estimated F_ROH_ ranged from 0.03 in YHC to 0.12 in NDC. For each of three ROH classes with different sizes (Small 0.5–1 Mb, Medium 1–5 Mb and Large >5 Mb), the numbers and total lengths of ROH per individual showed considerable differences across breeds. Moreover, we obtained 993 to 3603 ROH hotspots (which were defined where ROH frequency at a SNP within each breed exceeded the 1% threshold) among eight cattle breeds. Our results also revealed several candidate genes embedded with ROH hotspots which may be related to environmental conditions and local adaptation. In conclusion, we generated baselines for homozygosity patterns in diverse Chinese cattle breeds. Our results suggested that selection has, at least partially, played a role with other factors in shaping the genomic patterns of ROH in Chinese local cattle and might provide valuable insights for understanding the genetic basis of economic and adaptive traits.

## Introduction

Runs of homozygosity (ROH) are defined by contiguous homozygous segments where the two haplotypes transmitted from the parents are identical^1^. The occurrence of ROH within a population can be influenced by both reduced effective population size in the past and recent inbreeding. In addition, potential effect from genetic drift may also contribute to the formation of ROH. The availability of high-density SNP arrays has promoted the recent studies of regions of autozygosity. Initially, ROH analysis was utilized as a strategy to map recessive diseases genes in human^2,3^. Genome-wide analysis of homozygosity and their variation across individuals can offer important insights into genetic diversity and demographic history^4^. Abundance of ROH and their directional dominance on complex traits have been demonstrated in diverse human population^5^. Previous studies have investigated both genomic and geographic distribution of ROH and their contribution to population history. In human, recent studies suggested the total number and length of ROH per individual display considerable variation in worldwide human populations^6^, and the genomic distribution of ROH followed an obvious South to North gradient which was in consistent with European population history^7^.

ROH has also been widely utilized in farm animals to characterize population structure and demography history. Also, analyses of ROH can help to disclose the genetic relationships among individuals, measure the genome inbreeding level, and identify the region associated with economically important trait^8–20^. In cattle, Purfield *et al*. assessed the level of ROH in a wide range of daily and beef cattle breeds^11^, and their findings suggested different distribution patterns of ROH length may imply variations in breed origins and recent management among them. Extensive studies have been performed for evaluation of inbreeding and their negative effects on production and reproductive ability based on ROH in commercial daily cattle^15,16,18^. Also, several studies have been conducted to estimate inbreeding depression and investigate climatic adaptation in local cattle^14,21^.

Previous studies suggested the occurrence of ROH is not randomly distributed across the genomes, and ROH hotspots appear to be shared among individuals, which is probably caused by selection pressure^22^. The autozygosity across genome can help to improve the understanding of selection process and identify potential candidate genes associated with important traits in cattle^23–29^. The formation of ROH of various sizes (long vs. short) may reflect different evolutionary events on recent vs. ancient inbreeding^6,12^, with long ROH indicate more recent inbreeding, while short ROH suggest more ancient inbreeding. The investigation of these ROH segments can further improve our understanding of selection histories for special traits among diverse populations^23,24^.

Most of Chinese local cattle have been isolated in local feed environments, thus the potential homozygosity regions could be generated among them, which may also be influenced by adaptive selection and other factors in specific environment^30–32^. Despite several studies have conducted on analysis of population structures and admixture in Chinese local cattle^31,33^, no comprehensive study has been reported to explore the ROH pattern using high density SNP arrays. The aims of the present study were to (i) investigate genome-wide autozygosity patterns and genomic inbreeding level in Chinese local cattle; (ii) characterize profiles of ROH with different sizes and their gene contents with ROH hotspots; (iii) evaluate potential diverse selection based on the landscape of ROH across genome.

## Results

### Genomic ROH distribution

**Figure 1:**
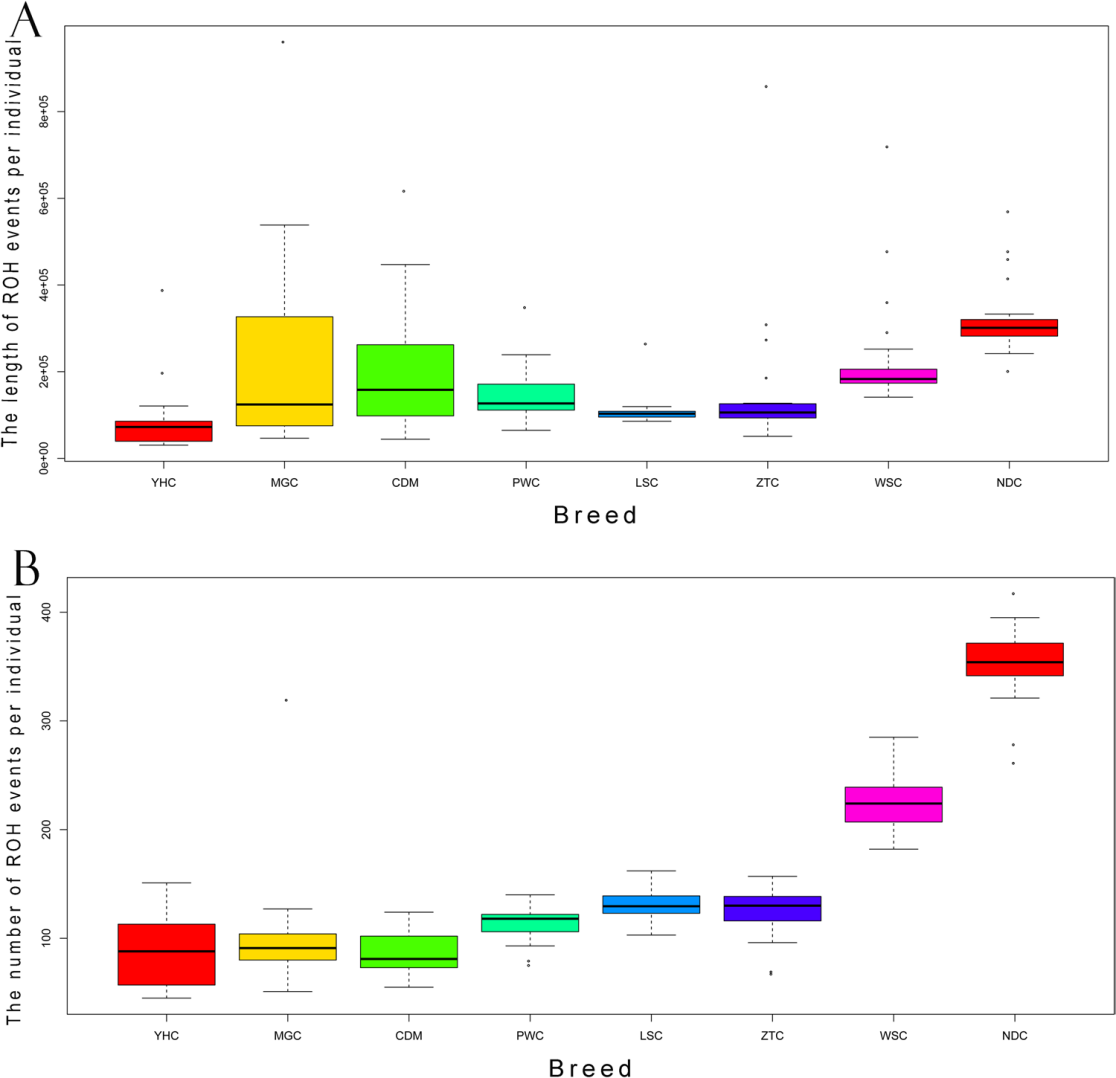
Individual patterns of ROH. The distributions of ROH statistics per individual for eight Chinese local cattle breeds, including YHC (n = 21), MGC (n = 21), CDM (n = 25), PWC (n = 23), LSC (n = 22), ZTC (n = 23), WSC (n = 21), and NDC (n = 23). Groups are divided based on geographic locations in China (North, Northwest, South and Southwest). (**A**) The length of ROH events per individual. (**B**) The number of ROH events per individual.

We identified a total of 27,358 ROH with an average of 153 per individual. The ROH size varied considerably from 0.5 to 66 Mb, with an average size of 1.22 Mb across all autosomes (Supplementary File 1: Table S1). The descriptive statistics of ROH and nonredundant ROH region per breed were presented in Table 1. Based on geographical locations, 8 breeds can be divided into 4 groups including North group (YHC and MGC), Northwest group (CDM), Southwest group (PWC, LSC and ZTC) and South group (WSC and NDC). We found cattle breeds from South and Southwest groups had the higher average ROH numbers, while cattle from North group had lower average numbers. The number of ROH per animal varied from 87 in CDM to 351 in NDC cattle (Table 1). Also, we observed the highest proportion of the genome covered by ROH in NDC (average ROHlength  per individual was 323 Mb, corresponding to ~11.54% of cattle genome) from South China, whereas the lowest was found in YHC (average ROHlength per individual was 87 Mb, corresponding to ~3.1%). As expected, the proportion of the genome coverage by ROH for each breed showed high correlation with their average observed heterozygosity (*Ho*) with *r* = 0.71. In addition, we found the numbers and sizes of ROH events per individual varied across populations. NDC had larger average numbers and sizes of ROH events than other breeds (Fig. 1).

**Table 1:** The descriptive statistics of ROH and ROH region for eight Chinese local cattle breeds.

	Sample Size	Total Number^a^	Average Number per ind	Total length (Mb)^b^	Average length Per ind (Mb)	ROH-region length (Mb)^c^	ROH-region number^d^	Genome Coverage^e^
YHC	21	1889	90	1821	87	1135	759	0.41
MGC	21	2156	103	4859	231	1906	423	0.68
CDM	25	2167	87	4900	196	2078	329	0.74
PWC	23	2601	113	3389	147	1675	721	0.60
LSC	22	2873	131	2397	109	1297	1011	0.46
ZTC	23	2861	124	3496	152	1800	650	0.64
WSC	21	4741	226	4935	235	1957	755	0.70
NDC	23	8070	351	7431	323	2129	770	0.76

Note:

^a^The total number of ROH events for each breed.

^b^The total length of ROH events across individuals for each breed.

^c^The length of ROH region for each breed.

^d^The number of nonredundant ROH region for each breed.

^e^The proportion of ROH coverage of genome for each breed.

### ROH region and inbreeding coefficients

ROH regions were determined by merging ROH identified across all individuals using previously published protocols implemented in BEDTools^34^. Totally, we identified 86 nonredundant ROH regions in Chinese local cattle, covering 2.47 Gb of the cattle genome (Supplementary File 2: Table S2). We observed the total length of merged ROH regions ranged from 1.13 Gb (~40.53% of genome) in YHC to 2.12 Gb (~76.02% of genome) in NDC (Table 1). Next, we estimated inbreeding coefficients within each population using three methods including F_ROH_, F_HOM_ and F_GRM_. The individual F_ROH_ values varied from 0.01 to 0.34, while the highest average F_ROH_ (0.12) was estimated in NDC. The F_GRM_ values were lower when compared to F_ROH_, which ranged from −0.07 to 0.36, whereas the coefficient for F_HOM_ ranged from −0.15 to 0.33. The highest correlation (*r* = 0.85, *P* < 2.2 × 10^−16^) was observed between F_ROH_ vs. F_HOM_, and the significant correlations were found for F_ROH_ vs. F_GRM_ (r = 0.78, *P* < 2.2 × 10^−16^) and F_HOM_ vs. F_GRM_ (*r* = 0.81, *P* < 2.2 × 10^−16^).

### Genomic patterns of homozygosity

**Figure 2:**
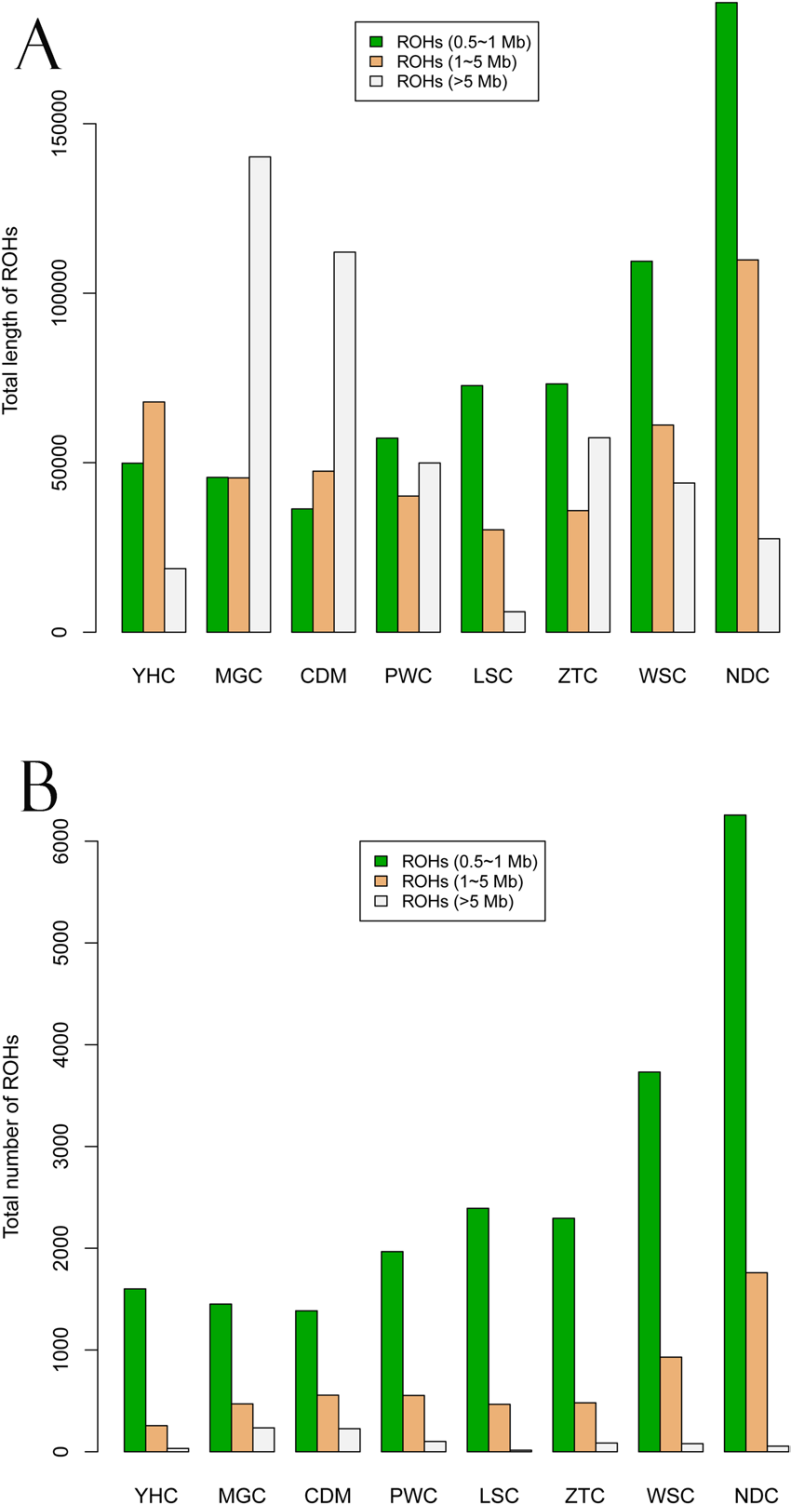
Total length and number of ROH in Chinese local cattle. (**A**) The total length of ROH belonging to three size classes including Small (0.5 to 1 Mb), Medium (1 to 5 Mb) and Large (>5 Mb) size classes for each of the different populations. (**B**) The total number of ROH belonging to three size classes.

To investigate the ROH pattern, we divided them into three size classes: A. Small (500 kb to 1 Mb), B. Medium (1 Mb to 5 Mb), and C. Large (>5 Mb), as described in the previous study^12^. The ROH distributions by total length and number for each group were shown in Fig. 2. The proportion of the genome in ROH of different lengths (thus inferred to be autozygous) varied clearly among breeds.

Our result showed two breeds (MGC and CDM) had the largest total lengths of Large ROH (>5 Mb, Fig. 2A) as compared to the other breeds, while the LSC had the smallest total length. Moreover, Small and Medium ROH were found to be predominant in the South group breeds (NDC and WSC) by their total lengths. Our results also revealed that Small ROH predominated across Southwest groups of Chinese local cattle (Fig. 2B). The total numbers of Small and Median ROH showed increasing trends for cattle from North to South groups.

**Figure 3:**
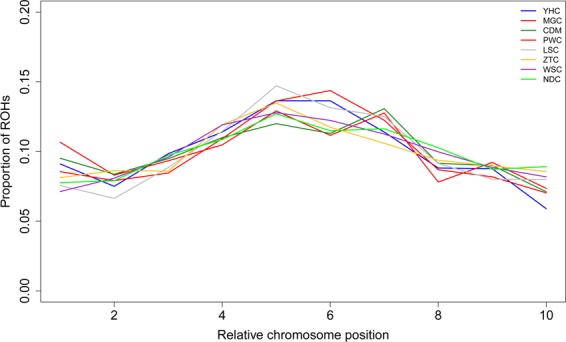
ROH distribution over relative chromosomal position in Chinese local cattle, and the distributions of ROH bins are averaged over all chromosomes.

As previously reported, the likelihood of ROH occurrence at a particular chromosomal position generally depends on the length of ROH^12^. We found that the distributions of ROH occurrence along the autosomes vary among eight breeds. The high proportion of ROH mostly appeared in the middle of the chromosomes with the low recombination rates, and much less towards the telomeric regions (Fig. 3). Additionally, NDC and WSC have high proportion of ROH across chromosomes when compared to other breeds.

### ROH profiles across populations

**Figure 4:**
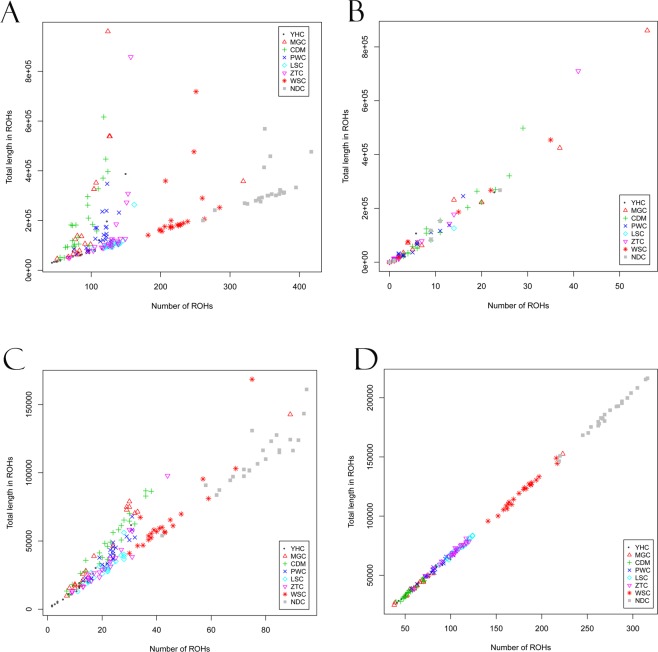
Evaluation of number of ROH and ROH total length for eight cattle populations. The number of ROH found for each individual genome (*x-axis*) is plotted against ROH total length (i.e. the length of Mb covered by ROH in each genome, *y-axis*). We analyzed cattle from YHC, MGC, CDM, LSC, PWC, ZTC, WSC and NDC. (**A**) All ROH; (**B**) size class Large (>5 Mb); (**C**) size class Medium (1 to 5 Mb) and (**D**) size class Small (0.5 to 1 Mb).

To study the demographic history, we firstly plotted the numbers of ROH per individual against the total length of them (Fig. 4A). We observed ROH profiles (length vs. number) displayed different patterns among eight breeds. Out of 8 cattle breeds, YHC showed a small number of ROH and short total length in ROH with most points locating in the left corner of the plot (Fig. 4A). MGC, CDM, ZTC, and PWC had most points in the higher left corner of the plot, indicating a small number of ROH, but a large sum of total ROH length. WSC and NDC were extreme cases with large numbers of Small and Medium ROH and large total ROH lengths. Also, we observed the different patterns of ROH when ROH were classified into three class by sizes, including Large (Fig. 4B), Medium (Fig. 4C) and Small classes (Fig. 4D). Our results suggested Medium and Large ROH showed large differences among individuals.

**Figure 5:**
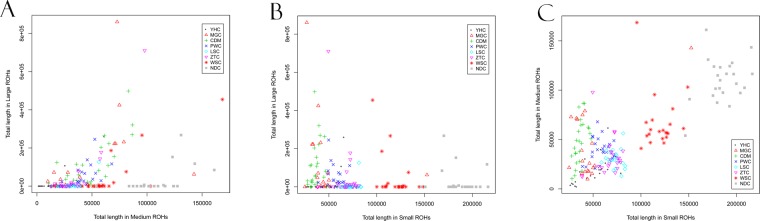
Comparison of total lengths of ROH per individual across size classes, including Small, Medium and Large. Pairwise correlations and their significance levels between the total lengths of Small, Medium, and Large ROH in individual genomes were estimated using Pearson correlations. (**A**) Large versus Medium (r = 0.72). (**B**) Large versus Small (r = 0.36). (**C**) Medium versus Small (r = −0.19).

To assess the genomic distribution patterns of total ROH length, we also conducted pairwise comparisons among the total lengths of three classes of Small, Medium and Large ROH in individual cattle genomes (Fig. 5). We found the total lengths of Small and Medium were highly correlated (r = 0.72, *P* < 2.2 × 10^−16^), while none of them is strongly correlated with the total length of Large (r = 0.36 with *P* < 5.36 × 10^−7^ and r = −0.19 with p < 0.0106, respectively).

### ROH involved with selection signatures

**Figure 6:**
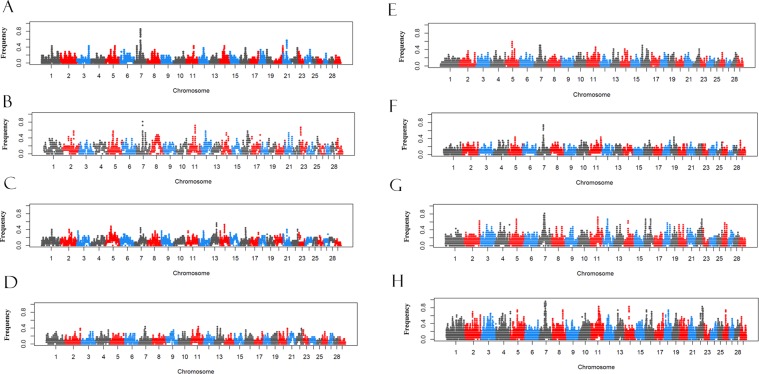
Manhattan plot of the distribution of runs of homozygosity (ROH) hotspots in the Chinese local cattle genome. The *x-axis* represents the SNP genomic coordinate, and the *y-axis* shows the frequency (%) of overlapping ROH shared among individuals. (**A**) YHC; (**B**) MGC; (**C**) CDM; (**D**) PWC; (**E**) LSC; (**F**) ZTC; (**G**) WSC; and (**H**) NDC.

To investigate effect of selection in diverse cattle, we explored the occurrences of ROH across genome. The frequency of a SNP locus (%) in those ROH was assessed for each breed, and then plotted against the position of the SNP along autosomes (Fig. 6). The threshold for each breed were 0.29, 0.48, 0.4,0.30,0.32,0.30, 0.57, 0.74 for YHC, MGC, CDM, PWC, LSC, ZTC, WSC and NDC cattle. Our results suggested that the occurrences of ROH varied clearly within the genome among diverse cattle. We observed 3603, 1542, 1055, 2195, 3216, 1839, 1444 and 993 hotspots at top 1% in YHC, MGC, CDM, PWC, LSC, ZTC, WSC and NDC cattle, respectively. Within the genomic regions with a high level of autozygosity under potential selection, we identified 382 unique genes across eight breeds. Among them, twelve genes including *CTNNA1*, *LRRTM2*, *SIL1*, *SNHG4*, *MATR3*, *PAIP2*, *MZB1*, *SPATA24*, *DNAJC18*, *ECSCR*, *TMEM173*, *UBE2D2* were shared across eight breeds. The DAVID annotations for these top annotated genes for each of eight breeds were presented in Supplementary File 3: Table S3. Most genes identified in North and Norwest group, including MGC and CDM, were enriched in lysozyme activity, cytolysis, defense response to Gram-negative bacterium and cell wall macromolecule catabolic process.

Among 382 unique genes, a total of 137 were identified in two and more populations. In YHC, two regions were detected on BTA7 and BTA21, including two genes *(ETF1* and *HSPA9*) at 51.5 Mb on BTA7 and three genes (*TMEM266*, *ETFA* and *ISL2*) at 32 Mb on BTA21. In MGC, we detected top three regions on BTA7, BTA11 and BTA23: the top region on BTA7 overlapped with six genes *CTNNA1*, *LRRTM2*, *SIL1*, *SNHG4*, *MATR3* and *PAIP2*, while no known gene was found within the regions on BTA11 and BTA23. Also, we observed the top three regions on BTA5, BTA13 and BTA14 in CDM. Among them, the top region on BTA5 contained genes *YEATS4* and *LYZ1*. We found 17 genes were included in the region ranging from 54.48 to 54.85 Mb on BTA13, while no known gene was found within the region on BTA14. In the LSC cattle, we observed one significant region on each of BTA5, BTA7 and BTA16, which overlapped with many genes and spanned 0.6 Mb, 7 Mb and 0.1 Mb, respectively. While in the ZTC, only one region surpassed the 50% occurrence of ROH which ranged from 51.6 to 52.7 Mb, containing the important gene like *CTNNA1*, *ISL2*, *DNAJC18*, and *SPATA24*. Finally, no significant region (>50% occurrence) was detected for the PWC in Southwest groups. For two breeds in South group, we observed that multiple regions on BTA2, BTA5, BTA7, BTA11, BTA16, BTA14 and BTA22 were shared by WSC and NDC within the regions of above 50% occurrences.

In addition, a total of 247 genes were identified in unique breed based on the occurrence of ROH, of those, 56, 9, 15, 69, 28, 38, 24, 8 genes were uniquely identified in YHC, MGC, CDM, LSC, PWC, ZTC, WSC and NDC, respectively.

## Discussion

Cattle represent important genetic resources that contribute to local economy. Many studies have been explored the ROH pattern and its associated inbreeding depression related to traits at the genomic level in multiple cattle population^8^. However, the ROH patterns and their distributions in Chinese local cattle remain largely unexplored. To our knowledge, this study is the first attempt to investigate the occurrence and distribution of ROH in Chinese local cattle using high-density SNP arrays. The high density SNP arrays are more sensitive for the determination of small segments, as previously reported^35^, while the BovineSNP50K array may underestimate the numbers of segments of 1 to 4 Mb in length.

We estimated inbreeding coefficients within each population using three methods including F_ROH_, F_HOM_ and F_GRM_. In general, the correlations among F_ROH_, F_HOM_ and F_GRM_ varied dramatically across studies. Our study revealed the relative high correlation (0.78~0.85) among them, and high correlations between F_ROH_ and the two other estimates of genomic inbreeding (F_GRM_ and F_HOM_) can be considered as an accurate estimator of the IBD genomic proportion^17^. On the other hand, several other studies have found a low to moderate F_GRM_-F_ROH_ correlation for dairy breeds^36,37^, while moderate to high correlations were reported in Holstein cattle and Jersey populations^15,38^. We suspected that these different results could be related to ROH type, sample populations and SNP array platforms.

In this study, we found clear differences in terms of total number and length of ROH in diverse Chinese local cattle, and the relationship between them varied considerably among individuals. This was consistent with previous studies with large-scale datasets in cattle, their findings suggested that ROH are often differentially distributed across breeds, and the patterns of ROH (number and length) may imply differences in breed origins and recent management^11^. The British Isles breeds including Hereford, Guernsey, Angus and Jersey cattle displayed the highest sum of ROH per animal compared other breeds, while African breeds displayed high variability in total number of ROH among breeds^11^.

A recent study revealed that the average total length of ROH ranged from 106 Mb in Piedmontese to 371 Mb in Brown cattle^36^, and their study suggested that dairy breeds have longer and larger size ROH than beef and dual-purpose cattle, and inbreeding is more recent in these dairy breeds. In our study, we found some breeds like WSC and NDC in South China displayed larger ROH size, which may reflect the effects of current inbreeding. We suspected that both breeds may be selected in isolated conditions with relative small effective population sizes. This finding also implied that continuing isolation and a reduced population size might play an essential role in the formation of ROH.

The distribution of ROH numbers and lengths can illustrate the genetic diversity of studied population. We also observed diverse pattern between the total number and length of ROH in Chinese local cattle, which were agree with the previous goat and sheep studies^20,28^. A large proportion of individuals with large number and length were identified in NDC and WSC (Fig. 4A). In contrast, for the Small ROHs in each individual, the total length of Small ROH in NDC and WSC mostly ranged from 100 to 250 Mb and none of them was less than 100 Mb (Fig. 4D). This finding reflected that both ancient and recent inbreeding had a critical impact on cattle genomes. This ROH pattern might be caused by the breeding isolation of these populations. Additionally, the NDC and WSC have relatively small populations and might have experienced bottlenecks as indicated by high fraction of the genome in ROH. On the other hand, YHC showed very low amounts of ROH (Fig. 4A). This is consistent with recent admixture in the individuals from YHC. Otherwise, we observed medium level ROH in terms of both total length and number of ROH in CDM and PWC. This probably reflects an absence of admixture in those breeds. The comparison for distribution of ROH numbers and lengths may be useful to demonstrate the genetic diversity among cattle breeds.

A recent study assessed the pattern of homozygosity and they found the average coverage of genome with autozygosity was 175 Mb in Gyr (*Bos indicus*) cattle^17^, while our study found the average value of ROH coverage per animal were 323, 235 and 231 Mb in NDC, WSC and MGC, respectively. We also observed the cumulative ROH length was dominated by an abundance of Small to Medium ROH in WSC as well as NDC, which implied either of them is an isolated breed based on a source population of substantial size. Abundance of ROH due to genetic isolation has also been reported in European cattle populations^13^ and Japanese wild boar^12^. The accumulation of large ROH in individuals of MGC and CDM could be caused by potential selection pressure for special environmental conditions of these populations, which has also been reported in Spanish local goat breeds^20^. Three ROH class (Large, Small and Medium) may be influenced via different processes, including recent inbreeding and recombination on different evolutionary scales^6^. Clustering ROH into different size classes make it possible to detect and interpret genomic difference among breeds. In addition, previous study reported that long ROH are mostly enriched in regions with deleterious mutations, and inbreeding can elevate the occurrence of rare recessive diseases that represent homozygotes for deleterious mutations^39^. Therefore, control the increase of inbreeding can effectively promote the implement conservation programs and maintain genetic diversity in local cattle breeds.

Also, pairwise comparisons between the total lengths of Small, Medium, and Large ROH in individual cattle genomes suggested the total lengths of Small and Medium ROH are highly correlated (*r* = 0.72, *P* < 2.2 × 10^−16^). Neither class Small or Medium is strongly correlated with the total length of Large ROH (*r* = 0.36 with *P* < 5.36 × 10^−7^ and *r* = −0.19 with *P* < 0.0106, respectively). Our study revealed that most of ROH belongs to the Short to Medium ROH categories. Compared with breeds from Southwest and South, our results suggested that two breeds (MGC and CDM) have been high inbred based on high coverage of Large ROH (Fig. 2A), probably due to a relatively small population size and close mating for each of two breeds. Because long homozygosity segments across chromosome can be interrupted by recombination events, Large ROH are usually generated by recent inbreeding, while Small/Medium ROH are indicative of more ancient relatedness. For instance, a previous study highlighted ROH preferentially occur in regions of decreased recombination activity^12^. And another study reported the existence of recombination hotspots across the cattle genome can influence ROH^11^. Our finding was consistent with these results, and the identified regions with ROH hotspots frequently coincided with those with lower recombination rates. Moreover, we also found the studied populations display different proportions of ROH across the chromosomes (Fig. 3).

The occurrence of ROH hotspots in genomic regions that harbor candidate genes may be involved in selection pressure in response to environmental condition. Previous study has investigated homozygosity in Polish Red cattle, which are characterized by high fertility and easy calving, good immunity and health, as well as high milk quality^29^. Their finding revealed four genes regulating signaling lymphocytic activation including *SLAMF1*, *CD48, Ly9* and *SLAMF3*. In our study, we found several genes within ROH hotspots, which have been previously reported under selection in cattle^40–45^. Among them, we identified Heat Shock Protein Family A (Hsp70) Member 9 (*HSPA9*) in seven Chinese local breed, this gene is immune-related genes, and also shows differentially expressed during bovine muscle development^46^. Previous studies suggested that *HSPA9* abundance in muscle had positive correlation with beef tenderness^47,48^. This regulation of *HSPA9* on meat tenderness is likely due to its anti-apoptotic effect, which prevents the formation of protein aggregates^49^. Moreover, we observed several genes like *CTNNA1*, *SIL1*, *PAIP2* in eight breed, and *ZBTB46* and *GMEB2* in CDM located within/near the ROH hotspots, which are also involved in selection in goat and sheep^50–52^.

To assesses the breed-specific differences in ROH occurrence related to selection, we investigated the genes within or near the ROH islands. In our study, we identified one gene named *EEF1A2 in* CDM, which was reported to be related to fatty acid and potentially regulate meat tenderness^53–55^. Several genes *SIRT6*, *CREB3L3* and *B3GNT5* were specifically detected in YHC cattle, which are reported related to energy meat quality, homeostasis and glycan synthesis^56–59^. Two genes *TCF12* and *TXNDC9* uniquely detected in LSC which were related to muscle lipid composition and marbling in beef cattle^60,61^. Moreover, we identified breed-specific genes (*BMP2*, *FGF9* and *CD14*) related to growth traits and immune process in PWC, WSC and ZTC, respectively^62–64^. Using the ROH approach, we detected many genes which show potential effects for important traits as reported in many previous studies. Our finding further supported that pattern of ROH can be utilized to explore genomic regions and genes under specific selection, and the shared ROH may potentially contain alleles associated with important traits. Despite many genes were identified in our ROH analysis, functional validation of them is still warranted in the future.

It was noted that we compared our settings to some other studies which used PLINK under its default settings^11,17,21^, and found out our settings generally agreed with the most of published studies using the Bovine HD SNP array. For example, Purfield *et al*. tested a minimum run length of 58 SNPs was needed to produce <5% randomly generated ROH and the maximum gap between two consecutive homozygous SNPs in a run was set at a 100 kb^11^. We found their options used was like ours, which slides a window of 50 SNPs, in one SNP intervals, across the genome estimating homozygosity. Although a previous simulation study has tested out ROH calling programs and their settings and reported the best program (PLINK) and its optimal settings^65^, the ROH calling methods and their settings are still challenging^11,35^, thus these results including ours should be interpreted by cautions. Also, to avoid bias and overestimation for ROH detection using PLINK, more options should be considered carefully according to the genome size and the number of SNPs genotyped.

In summary, our study revealed the occurrence of autozygosity varied largely across breeds in Chinese local cattle as different recent and ancient events. We further speculate that selection has partially contributed to landscape of ROH and their functions in the Chinese local cattle.

## Materials and Methods

### Ethics statement

All animal experiments were approved by the Chinese Academy of Agricultural Sciences (CAAS, Beijing, China). All animal procedures were performed in strict accordance with the guidelines proposed by the China Council on Animal Care and the Ministry of Agriculture of People’s Republic of China.

### Genotyped samples

The genome-wide SNP data of 179 samples from eight populations including Yanhuang (YHC), Menggu (MGC), Caidamu(CDM), Liangshan (LSC), Pingwu(PWC), Zhaotong (ZTC), Wenshan (WSC) and Nandan (NDC) were genotyped by Illumina BovineHD SNPs array, which was retrieved from our previous study^32^. The full names, associated abbreviation for each breeds and additional information on the locations of the sampling areas was presented in Supplementary File 4: Table S4. To minimize genetic relationships among samples, we removed closely related individuals if the PI-HAT value was greater than 0.25. Only autosomal SNPs passed following filters were used for subsequently analyses, and all selected samples displayed a genotype calling rate of more than 99%. Individuals with more than 5% of missing SNPs were removed from further analyses. Moreover, the SNPs were filtered with MAF < 0.05 (a minor allele frequency higher than 0.95) and geno <0.1 (only SNPs with a 90% genotyping rate or higher).

### ROH estimation

ROH were detected across autosomes for each individual using PLINK v1.07^66^. The ROH were defined by a minimum of 0.5 Mb in length to avoid short and common ROH that occur throughout the genome due to LD. The PLINK software detect homozygous segments by scanning along genotype for each individual. The following six criteria were considered to define a ROH: (i) a sliding window of 50 SNPs across the genome; (ii) the proportion of homozygous overlapping windows was 0.05; (iii) the minimum number of consecutive SNPs included in a ROH was 100; (vi) a required minimum density was set at 50 kb/SNP; (v) the minimum length of a ROH was set to 500 kb, the maximum gap between consecutive homozygous SNPs was 100 kb; and (vi) a maximum of two SNPs with missing genotypes and up to one heterozygous genotype were allowed in a ROH.

### ROH classification and inbreeding coefficient

ROH were divided into three classes based on length: 0.5–1 Mb, 1–5 Mb and >5 Mb and were identified as Small, Medium and Large, respectively. Three metrics were used to estimate levels of inbreeding: (1) F_HOM_ was assessed based on the proportion of homozygotes using PLINK v1.07 software. (2) F_ROH_ was calculated as described by McQuillan *et al*. (an individual’s summed ROH length was normalized by the length of the autosomal genome covered by SNPs)^67^. (3) F_GRM_ was estimated based on the variance of additive genotypes using GCTA 1.19.2 method according to Yang *et al*.^68^. Correlations of inbreeding coefficient for three methods were estimated using *cor.test* function in R v3.2.4.

### Statistical analysis of the genomic ROH across breeds

The correlations between the average proportion of the genome coverage by ROH and average observed heterozygosity (Ho) for each breed were estimated by *cor.test* functions in R v3.4.2. The ROH pattern based on the total number of ROH per individual and the total length of those ROH were measured for three classes. For analysis of distribution of ROH across genome, each chromosome was divided into 20 equal size segments, and their relative distribution over these chromosomal segments were calculated across breeds. All plots in current study were generated with the R programing (v.3.2.4).

### Genomic regions within ROH

To investigate genomic regions that were associated with occurrences of ROH within each breed, the fraction of SNPs in ROH was estimated based on the frequency of a SNP in them across individuals. ‘ROH hotspot’ was identified as a region of adjacent SNPs with an ROH frequency per SNP above the threshold of 1%. Genomic coordinates for all identified ROH regions were used to annotate genes using the UMD3.1 genome assembly. The function of these genes and GO terms were further assessed using DAVID Functional Annotation Bioinformatics Microarray Analysis platform^69,70^.

## Supplementary information

Supplementary files

Supplementary File1_Table S1, Supplementary File2_Table S2, Supplementary File3_Table S3, Supplementary File4_Table S4
