# Supplementary material for: Genomic Patterns of Homozygosity in Chinese Local Cattle
Source: Sci Rep. 2019 Nov 18;9:16977. doi: 10.1038/s41598-019-53274-3 (PMC6861314; doi:10.1038/s41598-019-53274-3)
Supplement: Supplementary file 1 — Supplementary files [file 41598_2019_53274_MOESM1_ESM.pdf]

Genomic Patterns of Homozygosity in Chinese Local Cattle

Lingyang Xu[1†,*], Guoyao Zhao[1†], Liu Yang[1], Bo Zhu[1], Yan Chen[1], Lupei Zhang[1], Xue Gao[1], Huijiang Gao[1], George E. Liu[2], Junya Li[1*]

[1] Laboratory of Molecular Biology and Bovine Breeding, Institute of Animal Sciences, Chinese Academy of Agricultural Sciences, Beijing, 100193, China.

[2]Animal Genomics and Improvement Laboratory, Department of Agriculture-Agricultural Research Services, Beltsville, Maryland, 20705, United States of America.

Supplementary files

Supplementary File1:

Table S1. The identified individual ROH across 179 individuals and ROH summary statistics per individual.

Supplementary File2:

Table S2. The nonredundant ROH regions in Chinese native cattle. ROH regions were determined by merging ROH identified across all samples within/across breed.

Supplementary File3:

Table S3. The DAVID annotations for the top annotated genes for each of eight breeds.

Supplementary File4:

Table S4. The full names, associated abbreviation for each breeds and additional information on the locations of the sampling areas.
